# Plug-and-Play *In Vitro* Metastasis System toward Recapitulating the Metastatic Cascade

**DOI:** 10.1038/s41598-019-54711-z

**Published:** 2019-12-02

**Authors:** Bing-Syuan Ni, Ching Tzao, Jen-Huang Huang

**Affiliations:** 1 Department of Chemical Engineering, National Tsing Hua University, Hsinchu, 30013 Taiwan; 2 Kuang Tien General Hospital, Taichung, 43303 Taiwan

**Keywords:** Lab-on-a-chip, Experimental models of disease

## Abstract

Microfluidic-based tumor models that mimic tumor culture environment have been developed to understand the cancer metastasis mechanism and discover effective antimetastatic drugs. These models successfully recapitulated key steps of metastatic cascades, yet still limited to few metastatic steps, operation difficulty, and small molecule absorption. In this study, we developed a metastasis system made of biocompatible and drug resistance plastics to recapitulate each metastasis stage in three-dimensional (3D) mono- and co-cultures formats, enabling the investigation of the metastatic responses of cancer cells (A549-GFP). The plug-and-play feature enhances the efficiency of the experimental setup and avoids initial culture failures. The results demonstrate that cancer cells tended to proliferate and migrate with circulating flow and intravasated across the porous membrane after a period of 3 d when they were treated with transforming growth factor-beta 1 (TGF-β1) or co-cultured with human pulmonary microvascular endothelial cells (HPMECs). The cells were also observed to detach and migrate into the circulating flow after a period of 20 d, indicating that they transformed into circulating tumor cells for the next metastasis stage. We envision this metastasis system can provide novel insights that would aid in fully understanding the entire mechanism of tumor invasion.

## Introduction

Tumor metastasis is a complicated and multi-step process involving tumor growth, local migration, intravasation, dissociation into the circulating blood or lymphatic system, and extravasation into the distal organs to complete the invasion-metastasis cascade, a process which is responsible for more than 90% of cancer-related mortality^1,2^. Current developments in cancer research have made cancer treatment possible by using cytotoxic-based treatments to successfully manage or reduce the size of most solid tumors, a process referred to as tumor shrinkage before cell metastasis reaches secondary sites. However, underestimation of the cancer cell invasion and metastasis, as well as misadministration of antimetastatic drugs, has made the full puzzle of tumor progression difficult to complete^3,4^. More evidence has been reported that tumor cells might have disseminated early to and established (micro)metastases in the secondary sites before a diagnosis is confirmed^5,6^. In addition, the success of tumor metastasis is strongly associated with metabolic rewiring that promotes the survival of disseminated cancer cells^7^. Therefore, these demonstrate the need for novel therapeutic strategies and antimetastatic drugs that can localize and prevent the spread of invasive cells.

To better understand the mechanisms that underlie cancer metastasis and to discover new therapeutic strategies and effective antimetastatic drugs, numerous models have been specifically established for preclinical research. Recently, *in vivo* studies have made progress in reconstructing earlier and more accurate predictive models, such as patient-derived xenografts (PDX) implanted in humanized mice or genetically engineered mouse models (GEMMs)^8^. Although these animal models have proven to be important tools for analyzing the complex interactions involved in the metastatic cascade, they are still limited because they introduce inconsistencies and poor reproducibility, and are time-consuming, labor-intensive, and lack high-throughput screening and real-time imaging^9^. Furthermore, some tumor models cannot even be established in PDX and used for tumor research. Therefore, an alternative platform is essential for prescreening and to improve understanding of the detailed mechanisms of the metastatic cascade and cellular interaction within the tumor microenvironment^10,11^.

Recent studies have shown that the tissue culture conditions can be precisely controlled and the cell microenvironment can be manipulated for drug screening by using microfluidic-based technology^12,13^. The advantages of microfluidic technologies include the following:

1. They can improve the transfer efficiency of nutrients and oxygen into the tissue, thereby enhancing cell viability for drug studies^14,15^.
2. They can maintain the integrity and viability of tissue in comparison to conventional cell culture methods^16^.
3. They can generate concentration gradients of administered drugs to enable the tissue to spatially experience varying drug conditions at the same time^16,17^.
4. They can be used to co-culture other cell lines in the same device so that interactions between the various cells can be observed^18^.
5. They can manipulate multiple sample reservoirs at the same time using dynamic flow^19,20^.

These tumor metastasis chips were developed to co-culture tumor and endothelial cells on either side of a microchannel^21,22^ or porous membrane^23,24^ to generate *in vitro* tumor microenvironment. They are also employed to observe the transendothelial ability of tumor cells using real-time imaging systems that allow precise control of microenvironmental factors within defined endothelial barriers. Other examples are described that use an *in vitro* metastasis chip to enable the study of the extravasation of human cancer cells through an endothelial barrier toward the secondary metastasis site^25,26^. Although there is increasing research focusing on therapeutic strategies used for interrupting individual cancer metastatic cascade that involves clonal proliferation, cell migration, or other invasions^27^, there is no model that adequately describes the entire metastasis process owing to the difficulty in recapitulating and connecting each of the required steps of metastasis. Moreover, it is still uncertain whether the progression of cancer relies on biochemical or biophysical responses such as interstitial flow and collagen properties^28,29^. These limitations impede the development of appropriate preclinical models that truly reflect a physiologically relevant metastatic mechanism that could be used to adequately validate a potential antimetastatic therapeutic agent.

**Figure 1:**
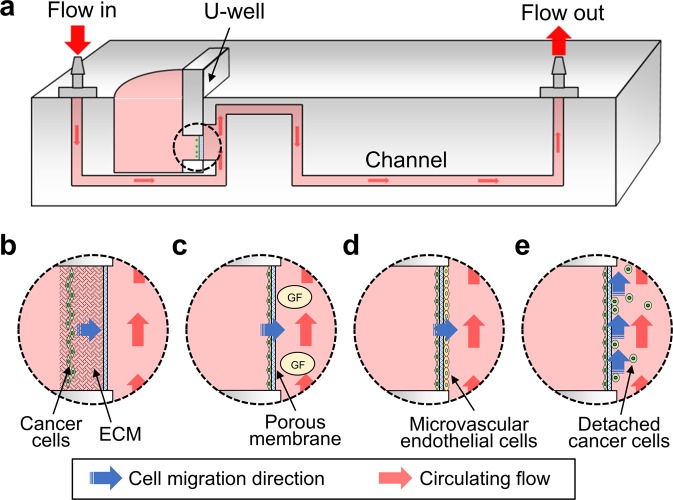
(**a**) Schematic diagram illustrates a cross-section view of the metastasis chip. The design allows for the evaluation of the metastasis capability of cancer cells at different stages using a plug-and-play design. The U-well can be removed and placed under a microscope to monitor the cell behaviors inside the region of the U-well circled with a dashed line: (**b**) Cancer cell migration through the ECM. (**c**) Cancer cell intravasation using growth factor (GF). (**d**) Cancer cell intravasation with a co-culture of microvascular endothelial cells. (**e**) Cancer cell detachment into the circulating flow.

**Figure 2:**
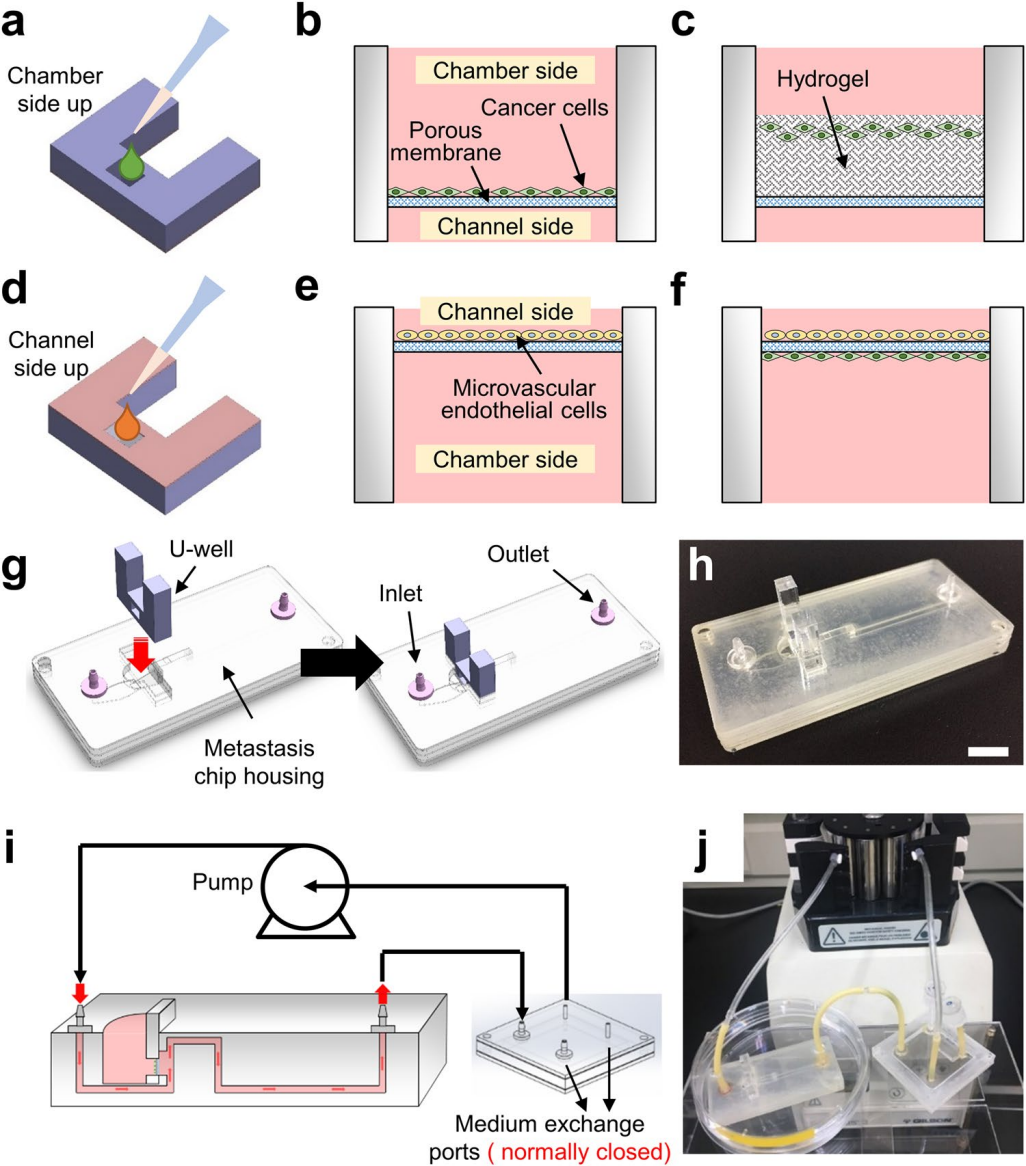
U-well and plug-and-play culture design allowing for culturing various types of cancer cells and performing diverse culture techniques in a simple fashion. (**a**) The cancer cells can be directly seeded on the chamber side of the U-well using a pipette. (**b**) Once the cancer cells are seeded, these cells can attach to the porous membrane to form a mono-cell layer after incubation. (**c**) The cell-laden hydrogel can be pre-mixed with and loaded into the U-well to generate a 3D culture environment. (**d**) The microvascular endothelial cells can also be seeded on the channel side of the U-well to investigate the influence of shear stress. (**e**) The attached microvascular endothelial cells on the channel side of the U-well. (**f**) The cancer cells can be seeded before or after the seeding of microvascular endothelial cells to form a co-culture environment. (**g**) The U-well can be plugged into the metastasis chip housing directly to complete the metastasis chip. The inlet and outlet of the chip housing are connected to the perfusion system. (**h**) An image of the metastasis chip. Scale bar = 1 cm. (**i**) Schematic setup of the metastasis system with a collection device and a peristaltic pump allowing for the collection of the circulating tumor cells under dynamic flow conditions. (**j**) An image of metastasis system setup.

To fulfill this requirement, an *in vitro* metastasis system that allows the culture of human cancer cells and complies with quantitative analysis to evaluate each stage of metastasis is demonstrated. The system builds upon a “plug-and-play” design that allows the cells to be seeded in advance in a U-shape insert (U-well), enabling the cells to grow in a 2D or 3D format and in culture along with other types of cells to reconstruct the tumor microenvironment. The cell-seeded U-well can be inserted into a microfluidic-based metastasis chip, providing a dynamic culture and perfusion environment for the cancer cells to invade the circulating flow (Fig. 1a). The U-well can be repeatedly pulled out of the metastasis chip for cell imaging under a microscope without affecting the entire setup of the system. These benefits allow the metastasis system to: (1) enable cell proliferation and migration in the 3D hydrogel matrix with biophysical induction (e.g. flow) (Fig. 1b); (2) achieve cell intravasation either by inducing biochemical induction (e.g. transforming growth factor-β1, TGF-β1) (Fig. 1c) or through the co-culture of human microvascular endothelial cells (Fig. 1d); and (3) investigate cell detachment into the circulating flow after long-term cell culture (Fig. 1e). These results suggest that the *in vitro* metastasis system developed in this study has the potential to become an alternative model for fully recapitulating the entire metastatic cascade and facilitating the investigation of antimetastatic therapeutic agents.

## Results and Discussion

### Multifunctional U-well enabling diverse cell culture environments

A two-sided chamber placed in the U-well was separated by a porous membrane to facilitate cell culture with diverse cell culture environments, including co-cultures, cultures in a hydrogel, and cultures within dynamic flow conditions. The porous membrane used in this study was obtained from a transwell culture insert, the most common *in vitro* assay for studying cancer metastasis, which enabled it to become a suitable carrier for culturing various types of cancer cells^12^. This replication of the cell culture environment can be accelerated to establish different stages of tumor metastasis through the use of a simple cell loading process. For example, a known density of cancer cells can be loaded directly into the chamber side well of the U-well using a pipette to complete the cell seeding process without concerning about the accumulation of air bubbles or non-uniform coverage in the metastasis chip during cell seeding (Fig. 2a). The loaded cells can then settle down and attach onto the chamber side of the porous membrane (Fig. 2b). By applying a similar loading process, a hydrogel or extracellular matrix (ECM) mixed with the cells can be transferred into the chamber side well to reconstruct the environment for cell migration (Fig. 2c). The culture of endothelial cells can also be performed by loading the cells on the channel side (opposite side) of the U-well (Fig. 2d) either with or without epithelial cells to generate a single culture (Fig. 2e) or co-culture (Fig. 2f), respectively.

To verify and to visualize the metastatic responses of cancer cells, adenocarcinomic human alveolar basal epithelial cells containing green fluorescent protein expression (A549-GFP) and human pulmonary microvascular endothelial cells (HPMECs) were chosen in this study. After loading of the cell into the U-well, the entire U-well can be placed in a 50-mm petri dish and cultured in an incubator, allowing the cells to uniformly attach and grow on the membrane (Fig. S1). It was verified that the A549-GFP cells and HPMECs required at least 6 h to completely attach to the porous membrane compared to the time to other microfluidic substrates such as polydimethylsiloxane (PDMS) which require 2 h to an overnight duration^30^. The seeding process can be viewed as a checkpoint to determine whether the cells have effectively attached to the membrane to proceed the further experiment. Once the cells attach to the membrane, the U-well can be turned to the other side to seed another type of the cells for co-culture purposes. Compared to the existing microfluidic-based culture chips that may easily introduce air bubbles in the microchannel during cell seeding or medium exchange^31^, these culture processes lend themselves to an easy-to-follow protocol for performing co-culture of cells, avoiding the uncertainties associated with cell seeding (e.g. non-uniform seeding), which might affect the subsequent metastasis experiment.

### Plug-and-play metastasis platform

The main innovation in this study is the plug-and-play technique, which allows for the U-well to be inserted directly in the metastasis chip housing to complete cell culture in static flow conditions (Fig. 2g,h). This plug-and-play feature allows the experimental setup time to be reduced and avoids initial culture failures during the cell seeding process when compared to other existing microfluidic metastasis chips. Furthermore, the air bubbles in the existing microfluidic-based culture chips may accumulate during long-term cell culture, altering the flow profiles and changing the cellular microenvironment. Therefore, the U-well was intentionally designed in a vertical format to prevent the accumulation of air bubbles in the channel in close proximity to the cell regions.

To mimic cellular microenvironments for the growth of tumor cells and to investigate the factors that may influence the metastatic cascade, it was necessary to connect the perfusion system to the metastasis chip to establish a dynamic culture condition. Typically, the metastasis chip was connected using syringe pumps, allowing for the generation of a stable culture environment. However, the continuous supply of fresh medium and removal of waste may lead to the loss of important biological messages secreted from the cells for regulating the tumor microenvironment that may reduce the efficacy of drugs circulated in the system^32^. Although some pumpless flow systems driven by hydrostatic pressure may be an alternative approach for providing a bidirectionally circulated flow using a programmable rocker, the medium must still be replaced every alternate day due to limited reservoir storage capacity and flow rate reduction owing to decreases in the internal pressure difference^33^. A conventional peristaltic pump was considered as an alternative approach to generate a stable and consistent circulating flow for long-term culture, although the external equipment was bulky and additional system setup time was required. To address these issues, a reservoir was installed between the peristaltic pump and the metastasis chip to increase the volume of the circulating medium. The metastasis chip housing was connected in advance, prior to the cell culture, and the entire system was perfused with the medium to remove air bubbles and establish a stable flow (Fig. 2I,j). For routine culture and maintenance of cells, the U-well could, therefore, be replaced for each experiment and the rest of the platform could still be used without changing the setup of the perfusion system. To maintain the consistency of circulating flow in the system for the growth of the cells, the flow velocity was set to 0.75 mm/s in the channel of the metastasis chip, corresponding to a wall shear stress of ~0.05 dyn/cm^2^ at the channel side of the U-well. This wall shear stress is within the range to promote tumor invasion and metastasis^34^. The inlet and outlet were connected to the same medium reservoir, so the entire system, including the U-well, could share the same medium during cell culture.

Unlike the cell observation when using the conventional transwell, the U-well is still required to be removed from the metastasis chip housing for routine microscope observation. Thus, human cervical cancer HeLa cells were cultured in the metastasis system to verify the capability and biocompatibility of the entire system. The cells were seeded on the chamber side of the U-well with a cell density of 4 × 10^4^ cells/mL and cultured at the flow velocity of 0.75 mm/s for 3 days so that the proliferation process of the cells can be monitored with time. Fig. S2a demonstrates that the HeLa cells were able to attach to the porous membrane of the U-well with 50% confluence on day 1 and kept proliferating on day 2 (Fig. S2b). After 3 days of culture, the cells reached 95% confluence, revealing the system is able to culture the cells in 2D format without contamination after the daily observation (Fig. S2c).

### Effect of hydrogel thickness and flow conditions on cell migration

Many studies have shown that the interstitial flow between the ECM of tissue can induce a pressure gradient that stimulates and guides tumor cell migration owing to the gene modification associated with epithelial-mesenchymal transition (EMT)^35–37^. To evaluate whether the metastasis platform developed in this study could also be applied to the study of the cell migration influenced by interstitial flow, a two-layer collagen matrix combined with cell-free and A549-GFP cell-laden collagen layers was constructed in the U-well. The cell-free collagen was prepared and loaded into the U-well to establish the desired thickness of the migration zone (*h*) for cell migration. After the cell-free hydrogel had solidified for 2.5 h, the cell-laden collagen with a cell density of 4 × 10^5^ cells/mL was added to the surface of the solidified hydrogel layer. A further 2.5 h duration was required to ensure completion of two-layer collagen matrix formation, allowing the cells to migrate in one direction toward the porous membrane The U-well containing the cells embedded in the collagen was plugged into the metastasis chip housing for the migration study. To verify whether the flow conditions (e.g. static or dynamic) and the thickness of the hydrogel could alter the behavior of cell migration, three different culture conditions and cell migration scenarios were established for comparison:

1. Cell migration in a static condition through the cell-free hydrogel with a controlled thickness of *h* ~ 765 µm (Fig. 3a).

**Figure 3:**
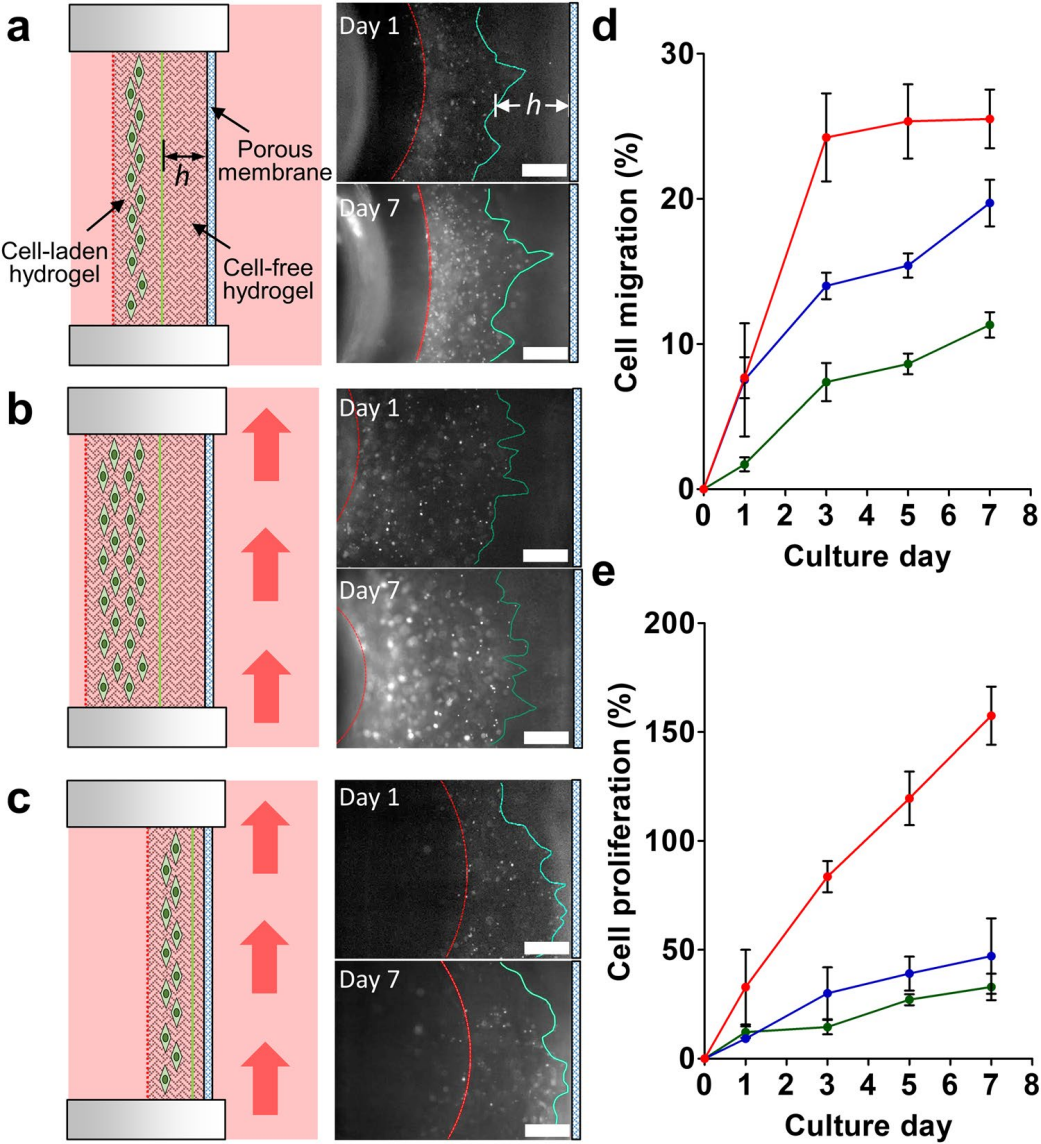
A549-GFP cell migration in the chip. (**a**) Cells migrate under static flow conditions through the cell-free hydrogel with a controlled thickness *h* ~ 765 µm. The red dashed line indicates the boundary between the cell-laden hydrogel and culture medium. The green line represents the leading front of the cells. Initially, it indicates the boundary between the cell-free and cell-laden hydrogels on day 0. Fluorescent images demonstrate the cells migration and proliferation after 1 and 7 days of culture. (**b**) Cells migrating through the thick cell-free hydrogel (*h* ~ 765 µm) under flow conditions. The red arrow indicates the flow direction. (**c**) Cells migrating through the thin cell-free hydrogel (*h* ~ 160 µm) under flow conditions. Scale bar = 500 μm. (**d**) Cell migration percentage under various conditions. Cells migrated through a thick hydrogel with static culture conditions (green), through a thick hydrogel with dynamic culture conditions (blue), and through a thin hydrogel with dynamic culture conditions (red). (**e**) Cell proliferation percentage under different culture conditions. Cell proliferated in a thick hydrogel with static culture conditions (green), in a thick hydrogel with dynamic culture conditions (blue), and in a thin hydrogel with dynamic culture conditions (red). N = 3.
2. Cell migration through a thick cell-free hydrogel (*h* ~ 765 µm) under flow conditions (Fig. 3b).
3. Cell migration through a thin cell-free hydrogel (*h* ~ 160 µm) under flow conditions (Fig. 3c). The flow velocity was set to 0.75 mm/s to achieve a circulating flow in the system.

The results demonstrated that the leading edge of the A549-GFP cell-laden matrix did migrate through the collagen in the direction of the porous membrane after 7 days of culture. The detailed fluorescence images of the cells are shown in Fig. S3. The quantitative data revealed that the cell migration percentage increased in all three culture conditions and the dynamic culture conditions (both in the thick and thin collagen layers) were able to enhance cell migration (Fig. 3d). Twice as many cells migrated (25%) within the thin collagen layer (*h* ~ 160 µm) than in the thick collagen layer (12%) under dynamic culture conditions after 3 d. Similarly, the cells embedded in the thin collagen layer demonstrated a 3-fold proliferation percentage after 7 days of culture compared to the cells cultured in the thick collagen layer (Fig. 3e). The cells cultured under static and dynamic flow conditions within the thick hydrogel layer demonstrated similar proliferation percentages during the culture process.

The flow velocity on the gel matrix side (or interstitial flow) was relatively low owing to the presence of a porous membrane barrier when compared to the microchannel side, leading to a low Péclet number (Pe = *u × h*/*D*_*s*_, where *u* is the flow velocity, *h* is the width of channel, and *D*_*s*_ is the diffusivity of a solute in the solution)^38^. In other words, the convective mass transport was much lower than the diffusion mass transport in the gel matrix side, suggesting that the mass transport through the hydrogel matrix in the U-well was merely dominated by the thickness of the hydrogel. This creeping fluid flow can then be expressed by introducing Darcy’s law^39^ as *v* = *k*Δ*p*/(*µL*), where *v* is the interstitial fluid velocity through the hydrogel perpendicular to the medium flow, *k* is the fluid permeability, Δ*p* is the pressure drop between the chamber side and the channel side of the U-well, *µ* is the viscosity of the fluid, and *L* is the thickness of hydrogel. When other parameters remain unchanged, the velocity of interstitial flow is merely based on the thickness of the hydrogel, which indicates the interstitial flow in a hin hydrogel can induce a higher cell migration than in a thick hydrogel^37^. Furthermore, it reveals that cancer cells exposed to dynamic flow conditions can migrate further than those exposed to static conditions^40^.

Furthermore, it was found that the cells in the thin collagen culture adopted a similar migration position (on the edge of the collagen matrix, Fig. 3c) that reached the membrane after 3 days of culture. It was assumed that the cells may begin to intravasate out of the collagen and attach to the membrane after 7 days of culture. To verify this assumption, the two layers of collagen in the U-well were carefully removed using a pipette. Unfortunately, no cells were found to have attached to the membrane or to have migrated across the membrane (Fig. S4). The results suggest that although approximately 25% of the cells within the thin hydrogel were able to migrate toward the membrane (Fig. 3d), no cells could escape from the collagen matrix and intravasate across the porous membrane after 7 days of culture.

Unlike conventional cell invasion assessments using thin layers of Matrigel of a few microns in thickness for cell penetration^41^, the collagen matrix used in this study may be too thick for cells to penetrate effectively to reach to the porous membrane.

### TGF-β1 activation and HPMEC co-culture-induced cell intravasation

**Figure 4:**
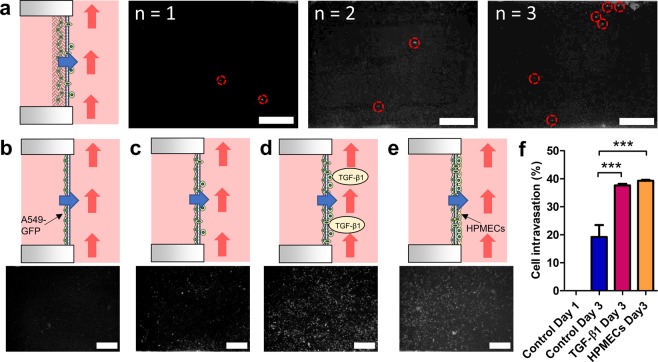
Intravasation of A549-GFP cells across the membrane in the chip. (**a**) Cell migration and intravasation from the collagen to the channel side of the U-well after 7 days of culture. Similar results were observed in three repeated experiments under a fluorescence microscope. Intravasated cells are enclosed in red circles. (**b**) A549-GFP cells were cultured in a monolayer condition for 1 d. The fluorescent image demonstrates no cells intravasated across the membrane. (**c**) A549-GFP cells were cultured for 3 d and began to intravasate. (**d**) A549-GFP cells were cultured for 3 d with TGF-β1 treatment, triggering more cells to intravasated. (**e**) A549-GFP cells were co-cultured with HPMECs for 3 d, enhancing the cell intravasation. (**f**) Cell intravasated percentages for the A549-GFP in the metastasis chip. ***P < 0.001. All cells on the chamber side of the U-well were removed to avoid the interference. Scale bar = 500 μm.

To further investigate whether the metastasis chip would allow cell migration and intravasation within the same setup, only one cell-laden collagen layer (thickness ~ 1000 µm) embedded with 4 × 10^5^ cells/mL of A549-GFP was loaded on the U-well and cultured for 7 d. After removing the collagen from the U-well, approximately 2 to 6 cells were observed to have penetrated the porous membrane to complete the intravasation within three different experimental setups (Fig. 4a). This result confirmed that the intravasation rate of A549-GFP cells was relatively slow, suggesting that a monolayer culture (no hydrogel) of A549-GFP cells might be a suitable approach for quantifying cell intravasation potential.

It is worth pointing out that an *in vitro* tumor microenvironment can be mimicked by culturing multiple cell types to generate complicated physical and biological cell-cell interactions, enabling the regulation of the invasion and metastasis of cancer cells through molecular signaling^42^. Recently, TGF-β1 has been shown to play a critical role in tumor progression and metastasis^43–45^, while a co-culture of microvascular endothelial cells is able to promote the growth of cancer cells^46^. Therefore, to quantify the actual intravasation rate of the cancer cells, the A549-GFP cells were cultured directly on the porous membrane in the U-well with a seeding density of 1.6 × 10^5^ cells/mL. For the co-culture experiment, HPMECs were seeded on the channel side of the membrane with a seeding density of 4 × 10^5^ cells/mL and cultured for 1 d prior to seeding with the A549-GFP (Fig. S5). To verify the cellular signaling acting on the metastasis chip, 5 ng/mL (0.4 nM) of the recombinant human TGF-β1 (R&D Systems, USA) was added to the culture medium on a daily basis until the end of the experiment^45^. After seeding for 1 d, the U-wells were inserted into the metastasis chip housing, which was connected to a perfusion system to generate the dynamic and circulating flow environment. It was found that no A549-GFP cells were observed on the channel side of the U-well on day 1 (Fig. 4b) but were able to penetrate the membrane to the channel side after 3 days of culture (Fig. 4c). It was also observed that cells cultured with TGF-β1 (Fig. 4d) and co-cultured with HPMECs (Fig. 4e) demonstrated the same trend, leading to an intravasation process after 3 days of culture. In comparison with that of the control, quantitative results showed that the cellular intravasation percentage significantly increased from 19.19 ± 4.25% to 37.62 ± 0.57% when the cells were treated with TGF-β1 and increased to 39.27 ± 0.35% when they were co-cultured with HPMECs, supporting the hypothesis that both growth and culture conditions can induce intravasation of the A549-GFP in the metastasis chip (Fig. 4f).

### Cell dissociation into the circulation system

**Figure 5:**
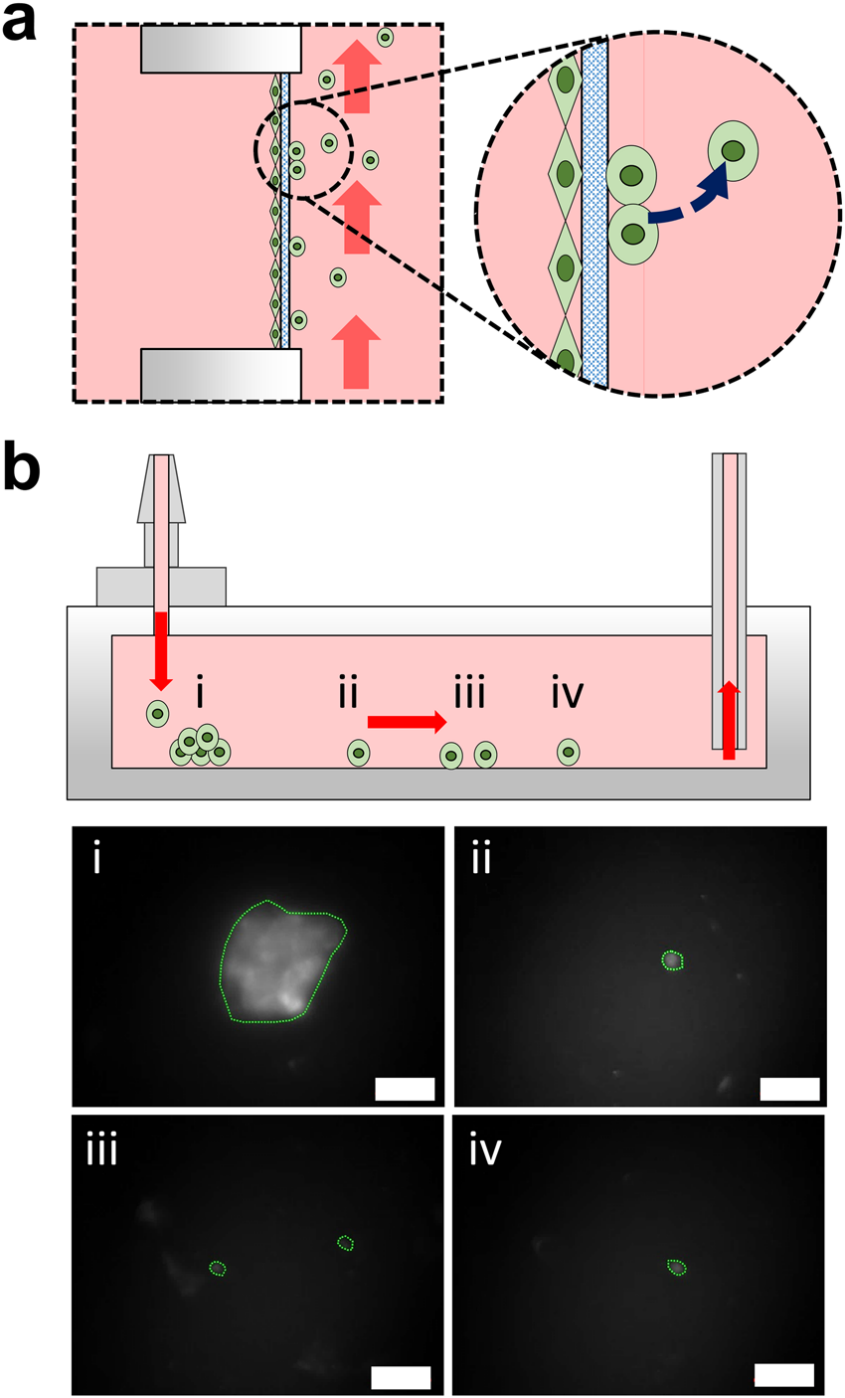
Dissociation of A549-GFP cells from the primary site. (**a**) The schematic demonstrates the intravasated cells detaching from the porous membrane and migrating with the circulating medium flow. (**b**) The cell collector connected after the metastasis chip can ensure the collection of dissociated cells. The sedimentation locations of detached cells in the cell collector were imaged after 20 days of culture. Cells are circled in green. Scale bar = 500 μm.

Tumor metastasis is a series of dynamic pathophysiological processes, involving cell migration, intravasation, detachment, transportation, and extravasation. Most of the *in vitro* tumor metastasis research focuses merely on studying tumor cell penetration through the endothelial monolayer into the blood flow (intravasation)^21^ or transendothelial migration into surrounding tissue (extravasation)^25^. The missing element of *in vitro* metastasis studies is the study of the detachment of tumor cells from the endothelium into the circulatory flow. This is also critical to understanding the full metastatic cascade process. These dissociated tumor cells in the blood stream, also called circulating tumor cells (CTCs), are an emerging multifunctional biomarker for the predictive, intermediate, and pharmacodynamic endpoints of drug responses^47^. To investigate whether the intravasated cells can dissociate from the primary site into the circulating flow, the A549-GFP cells were seeded in the U-well without embedding them in a collagen matrix owing to the slow intravasation process in the 3D format mentioned previously (Fig. 5a). The experimental duration was extended until the A549-GFP cells were observed either in the medium or cell collector (medium container). During the period of cell culture, the medium was first changed on day 5 and then every 3 d thereafter. The cell collector was observed every 3 d before replacing the medium. Furthermore, the medium removed from the container was filtered using a porous membrane (pore size = 5 μm) to collect any A549-GFP cells that were present in the circulating medium.

After 17 days of culture, live A549-GFP cells were collected on the filter membrane but no cells were observed in the cell collector, indicating that the cells had begun to dissociate from the primary site into the circulating flow (Fig. S6). Later, several live A549-GFP cells were found in the cell collector after 20 days of culture. The locations of the deposited cells are illustrated in Fig. 5b. Furthermore, cell aggregates were found in the corner of the cell collector, indicating that intravasated cells dissociated into the circulating system in both single cellular form (similar to CTCs) and also in multicellular cluster form, similar to circulating tumor microemboli. However, many of the cell debris was collected from the circulating medium and only one single live cell was found (Fig. S7). The preliminary finding demonstrates that most of the cells that dissociated from the channel side of the porous membrane were dead cells once they entered the circulating system. Although it is not clear whether these cells were dead before or after the dissociation process, the live single cells or cell aggregates found in both the circulating system and cell collector revealed that the late step of tumor metastasis could be established and monitored in the metastasis system developed in this study.

Although the A549-GFP cell line is ideal for monitoring cell behaviors such as proliferation and migration in real time, quantitative analysis results indicated that this cell line was not appropriate for fully studying the metastatic steps from cell proliferation, migration, intravasation, and dissociation into the circulating system. Other more aggressive cell lines or culturing the cells for even longer periods of time may be used to observe the full metastatic cascade in future experiments.

### Comparison with other *in vitro* metastasis models

*In vitro* tumor models based on microfluidic technology have emerged recently as new tools for antimetastatic drug discovery^31^. Compared with the gold standard approach — the transwell model, the microfluidic-based models have successfully recapitulated the key steps of metastatic cascades, including angiogenesis^48^, proliferation^49^, migration^50^, intravasation^21^, and extravasation^25^ because they allow for the precise control of fluidic flow and the 3D microenvironment for the growth of tumor cells. However, not all of the microfluidic tumor models are capable of recapitulating the entire metastatic cascade because tumor formation and metastasis is a long process. Table 1 expresses the advantages and disadvantages of the transwell model, the current microfluidic-based metastasis models, and our system setup with regards to operational performance and downstream analysis. The results in this study reveal that our model not only reflects the major features of the transwell model which can be used to seed and culture cells in a simple fashion but also combines the advantages of a microfluidic-based model that can introduce a dynamic flow to recapitulate the physiologically relevant conditions.

**Table 1:** Advantages and disadvantages of current *in vitro* metastasis models.

	System setup	Performance	Post analysis
Transwell model	Pros	Pros	Pros
	• Easy to operate	• High throughput	• Easy to recover the cells
	• No instruments required	• No air bubble formation	
	• No tubing and connection		Cons
	• Cell seeding is easy	Cons	• Additional transfer step required for observation
	Cons	• Frequent medium change for long term culture	
	• Static culture	• Gravity influence	
	• No physiologically relevant	• Can mimic only one step of the metastatic cascade	
Current microfluidic-based metastasis model	Pros	Pros	Pros
	• Dynamic culture	• Real-time monitoring	• Compatible with microscope
	• Co-culture	• Can generate a concentration gradient	Cons
	• Physiologically relevant	• Can mimic 1–2 steps of the metastatic cascade	• Difficult to recover the cells
	Cons	Cons	
	• Fabricated from PDMS	• Low throughput	
	• Requires trained personnel to operate and assemble	• Air bubble formation can ruin the experiment	
	• Requires a perfusion pump and other instruments		
	• Requires more attention for cell seeding		
This work	Pros	Pros	Pros
	• Fabricated from acrylic and PET	• Can perform long-term culture	• Easy to recover the cells
	• Easy to operate using a plug-and-play design	• No air bubble formation	• Can collect circulating cells
	• Cell seeding is easy	• Real-time monitoring	Cons
	• Dynamic culture	• Can mimic almost the entire cascade	• Additional transfer step required for observation
	• Co-culture	Cons	
	• Physiologically relevant	• Requires a medium reservoir for long term culture	
	Cons		
	• Requires a perfusion pump		
	• Requires a tubing connection		
	• Additional step for cell seeding		

In the context of the current microfluidic-based metastasis model, we remark that polydimethylsiloxane (PDMS), which is a standard manufacturing material for fabricating microfluidic devices, can only be used with specific antimetastatic drugs^51^. In addition, air bubbles can become trapped in the microfluidic-based model during long-term culture, making retrieval of cultured cells for downstream analyses challenging (e.g. genetic analysis, recovery of aggressive cells). Our model overcomes these limitations because it is fabricated with biocompatible and drug-resistant plastics (e.g. acrylic, polyethylene terephthalate (PET)). Moreover, its plug-and-play feature make the following possible: (1) Advanced cell seeding and culture to ensure better cell attachment to the culture surfaces without requiring additional seeding procedure; (2) simple and easy system setup that reduces the required operation time; (3) the co-culture and 3D culture of cells; (4) cell embedding in two-layer collagen matrices with controlled thicknesses to quantify cell migration; and (5) easy monitoring of cellular growth within the same sample without having to dismantle the device. Although an additional U-well transfer step is necessary for cell observation, it provides an additional opportunity to recover cells for downstream analyses similar to the transwell model.

## Conclusion

Through the use of the microfluidic-based metastasis system developed in this study, a plug-and-play *in vitro* model of a cell culture environment was successfully demonstrated along with its ability to trigger cancer cell migration, proliferation, intravasation, dissociation, and adhesion to a remote site to progressively mimic the metastatic cascade. It was also demonstrated that the metastasis system connected to a peristaltic pump and a medium reservoir enabled the dynamic culture of cancer cells, allowing for the reconstruction of a physiologically relevant model for understanding the mechanism of the metastatic cascade when TGF-β1 is added or a co-culture with HPMECs is performed. The integration of other U-wells into the metastasis chip housing also gives this system the advantages of the transwell model, such as easy operation and no air bubbles formation, allowing researchers to monitor the dissociation of cancer cells entering the circulating system. This model enables the study the interplay of other cell lines (e.g. immune cells) for further applications, such as the tumor immune microenvironment, and to establish an *in vitro* extravasation model in future designs by integrating another culturing chambers for the growth of healthy cell lines, such as osteoblast cells^52^, to investigate the invasion of cancer cells to secondary sites in the dynamic culture environment. We envision that the current metastasis system combined with the extravasation model has the potential to recapitulate more physiologically relevant conditions, enabling further understanding of the entire mechanism of tumor invasion.

## Methods

### Design and fabrication of plug-and-play metastasis chip

The goal of this study was to establish a recapitulating tumor microenvironment for investigating the tumor metastatic cascade using an easy setup procedure and a simple operational process. The metastasis chip consisted of a chip housing and a U-well to realize a plug-and-play approach. The chip housing was composed of 12 patterned plastic layers that could be defined into 5 groups based on functionality, allowing for connection of a pump and tubing, enclosing of the circulating medium in the channels and chambers, redirection of the flow in both vertical and horizontal directions, and mounting of the U-well for cell culture (Fig. S8a). The rectangular shape of the insertion groove (18.68 × 4.56 × 4.80 mm, width × height × depth) in the metastasis chip housing was designed to allow a tight fit for the U-well so that the medium could flow past the exposure window (5.0 × 3.5 mm) next to the insertion groove without leakage and associated loss of medium (Fig. S8b). On the other side of the insertion groove, a semicircular groove with a 3.5 mm radius and 7.5 mm depth served as a storage chamber, allowing for the storage of additional medium for cell growth.

The U-well contained two wells in both the chamber side and channel side layers that were separated by a porous membrane (Fig. S8c). The dimensions of the well on the chamber side were 4.0 × 2.6 × 3.0 mm and on the channel side were 4.0 × 2.6 × 0.25 mm. The porous membrane was cut from a transwell culture insert (pore size = 8 µm, Cat. No. 3428, Corning) and was sandwiched between the chamber side and the channel side layers using biocompatible adhesive tapes (Cat. No. 9122, 3 M Company) to provide cell seeding surfaces and to enable the transportation of nutrients and waste through the porous structure.

The medium reservoir was designed to allow the storage of ~4 mL of the medium so that the medium could be replenished every 3 d to reduce the impact of cell signaling losses incurred during medium replacement. The reservoir included two inlets and two outlets (Fig. S9). An inlet and outlet were connected to a pump for recirculating the medium, while another inlet and outlet were closed to maintain the balance of pressure until the medium needed to be replaced or until cytokines needed to be added to the system. The reservoir could also serve as a bubble trap to reduce bubble accumulation in the flow system. For long-term metastasis experiments, the reservoir could be used as a cell collector to trap dissociated cancer cells from the flow stream.

All of the devices used in this study were fabricated using a layer-by-layer stacking technique that had been previously developed^53^. Briefly, the devices were designed using the Solid Edge 2D software package (ST9, Siemens PLM Software). Each layer of the device was made by cutting pre-laminated polymeric sheets using a CO_2_ laser cutting machine (PLS6.75, Universal Laser System). The pre-laminated polymeric sheets were prepared by combining biocompatible adhesive tape with sheets of acrylic (1.5 and 3 mm thick, Formosa Idemitsu Petrochemical Corporation) or PET (0.25 mm thick, Formosa Idemitsu Petrochemical Corporation). After cutting, each layer was aligned using the alignment holes and assembled using a seam roller to complete the device assembly. Polycarbonate tubing adaptors (BDMR210–9, Nordson MEDICAL) were inserted in the chip and fixed with epoxy glue to complete the inlet and outlet connections of the chip. The outlet of the medium reservoir was constructed using a rigid polyetheretherketone (PEEK) tube (with 1/16” OD, 0.04” ID and 2 cm in length, Cole-Parmer) inserted in close proximity to the bottom of the reservoir chamber to allow complete drainage of the medium.

Each device was sterilized for at least 3 h using a 5% hydrogen peroxide solution in deionized (DI) water. They then underwent three washes using DI water followed by overnight UV irradiation in a cell culture hood to remove the remaining hydrogen peroxide. The connection tubing (PharMed BPT tubing with 1/16″ ID and 1/8″ OD, Cole-Parmer) was autoclaved prior to being used in the experimental setup.

### Cell culture and collagen preparation

A549-GFP cells (Cat. No. AKR-209, Cell Biolabs) and HeLa cells (gift from Prof. Yu-Chen Hu, National Tsing Hua University) were maintained in high glucose Dulbecco’s modified Eagle’s medium (DMEM) (Cat. No. SH30022.01, HyClone, GE Healthcare Life Sciences) containing 10% fetal bovine serum (FBS), (Cat. No. SH3007103HI, HyClone, GE Healthcare Life Sciences), and 1% Penicillin/Streptomycin Solution (P/S) (Cat. No. SV30010, HyClone, GE Healthcare Life Sciences). After a period of 3 to 5 d, when the cells reached 60–80% confluence, the cells were re-suspended in culture medium using 0.25% trypsin-EDTA (Cat. No. SH30042.01, HyClone, GE Healthcare Life Sciences) for seeding purposes. The seeding density of the cells was set to 1.6 × 10^5^ cells/mL for the 2D cell culture and 4 × 10^5^ cells/mL for the 3D cell culture in the hydrogel unless otherwise stated. HPMECs (Cat. No. 3000, ScienCell) were maintained in an endothelial cell medium (Cat. No. 1001, ScienCell) containing 5% FBS (Cat. No. 0025, ScienCell), 1% P/S, and 1% endothelial cell growth supplement (Cat. No. 1052, ScienCell). For co-cultured A549-GFP and HPMECs, the medium was prepared as a mixture of 50% DMEM medium and 50% of the completed endothelial cell medium. Both the A549-GFP and HPMECs were cultured in this medium mixture for one passage to make sure that the cells maintained their functionality prior to the co-culture procedure. For preparation of the cell-laden hydrogel, 3.5 mg/mL of type I collagen (rat tail tendon collagen, Cat. No. 354236, Corning) was adjusted to a pH of 7.0 with 0.1 M sodium hydroxide solution and mixed with the cells, followed by 2.5 h storage at 37 °C in an incubator for solidification to occur.

### Quantification of proliferated and migrated cells

The U-well could be removed from the metastasis chip housing, placed on the 50-mm perti dish, and transferred to the stage of the microscope to monitor the growth of the cells (Fig. S10a). The U-well was oriented vertically so that the images could be taken through the bottom side of the U-well (Fig. S10b) under an inverted fluorescence microscope (Eclipse TS100 with C-SHG light source, Nikon) connected to a digital camera system (LeadView 2800AM-FL, Leader Scientific). All of the images were obtained using the same exposure time to standardize the fluorescent intensity. The cell images were analyzed using ImageJ software (NIH) to obtain information on cell proliferation and migration in the hydrogel within a specific region of interest (ROI) (Fig. S10c). The number of cells could be quantified without post-staining owing to the green fluorescent protein (GFP)-derived signal in the cells. The cell migration images (Fig. S10d) could be evaluated based on Eq. (1) below^54^.

1 $${\rm{Cell}}\,{\rm{migration}}\,( \% )=\frac{{S}_{x}-{S}_{0}}{{S}_{0}}\times 100$$

where the cell migration percentage is the change in the area occupied by the cells at day *x* within an ROI. *S*_0_ is the fluorescent area measured on day 0 and *S*_*x*_ is the fluorescent area measured on day *x* within an ROI. The cell proliferation data were obtained by evaluating the fluorescent intensity change within an ROI (Fig. S10e). This can be described as in Eq. (2) below:

2 $${\rm{Cell}}\,{\rm{proliferation}}\,( \% )=\frac{{I}_{x}-{I}_{0}}{{I}_{0}}\times 100$$

where *I*_0_ is the fluorescent intensity measured on day 0, and *I*_*x*_ is the fluorescent intensity measured on day *x* within an ROI.

### Quantification of intravasated cells

Cells intravasated through the porous membrane could be monitored in the U-well. This assessment followed a similar procedure to the cell invasion assessment in the transwell. Briefly, the U-well was removed from the metastasis housing after culture. Non-intravasated cells on the chamber side of the porous membrane were removed with a sterile cotton-tipped applicator (Fig. S11a). Intravasated cells remaining on the U-well were observed under an inverted fluorescence microscope and analyzed using the ImageJ software package. The percentage of cell intravasation could be calculated using Eq. (3) below:

3 $${\rm{Cell}}\,{\rm{intravasation}}\,( \% )=\frac{{N^{\prime} }_{x}}{{N}_{x}}\times 100$$

where *N*_*x*_ is the area of cells measured on day *x* before removal (Fig. S11b) and *N*_*x*_′ is the area of cells measured on day *x* after removal (Fig. S11c) using a cotton-tipped applicator within an ROI.

### Analysis of circulating cancer cells

The cells that dissociated from the primary site in the U-well were collected in the medium reservoir (also called the cell collector) connected to the metastasis chip. The medium reservoir could be disconnected from the perfusion system by clamping the inlet and outlet tubing using binder clips. The collected cells in the medium reservoir could be monitored using fluorescence microscopy owing to the GFP (green fluorescent protein)-derived signals in the cells.

### Statistical analysis

Data were obtained from three independent experiments and analyzed using one-way ANOVA by GraphPad Prism (Version 5.0, GraphPad software). Results were considered significant when *P < 0.05, **P < 0.01, and ***P < 0.001.

## Supplementary information

Supplementary information
