# Supplementary material for: Plug-and-Play In Vitro Metastasis System toward Recapitulating the Metastatic Cascade
Source: Sci Rep. 2019 Dec 2;9:18110. doi: 10.1038/s41598-019-54711-z (PMC6889311; doi:10.1038/s41598-019-54711-z)
Supplement: Supplementary file 1 — Supplementary information [file 41598_2019_54711_MOESM1_ESM.docx]

**Supplementary information**

Plug-and-Play In Vitro Metastasis System toward Recapitulating the Metastasis Cascade

Bing-Syuan Ni^1^, Ching Tzao^2^ and Jen-Huang Huang^1*^

^1^Department of Chemical Engineering, National Tsing Hua University, Hsinchu, 30013, Taiwan

^2^Department of Medicine, Taipei Medical University, Taipei, 10031, Taiwan


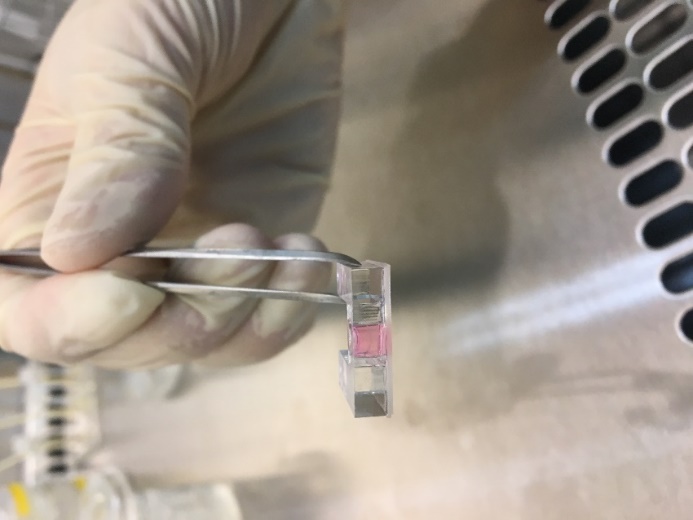

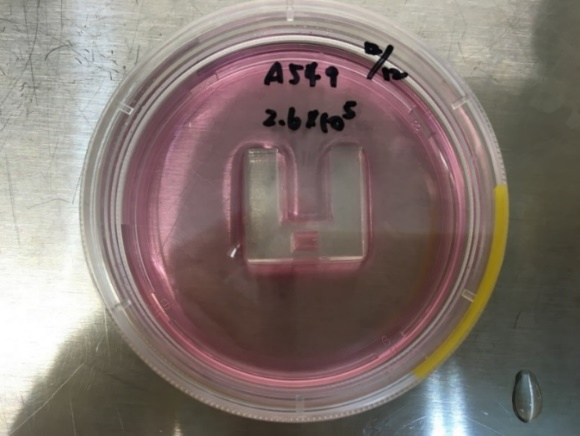


**Figure S1**. U-well can be transferred using forceps and placed within the 50-mm petri dish containing the culture medium after cell seeding. The cells in the U-well can uniformly attach to and grow on the porous membrane prior to installation on the metastasis chip.


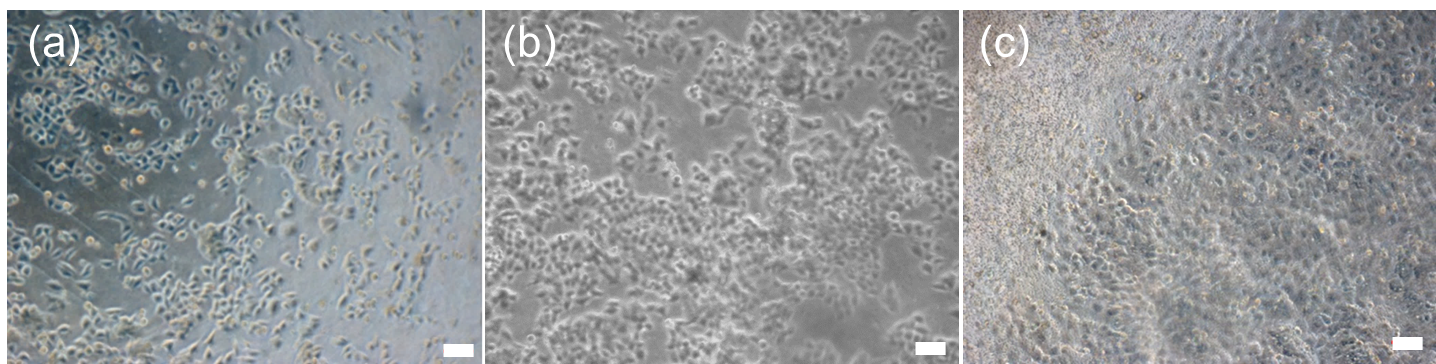


**Figure S2.** Proliferation of HeLa cells in the metastasis chip. The cells were seeded on the chamber side of the U-well with a cell density of 4×10^4^ cells/mL and cultured at the flow velocity of 0.75 mm/s on (a) day 1; (b) day 2; (c) day 3. Scale bar = 100 µm.


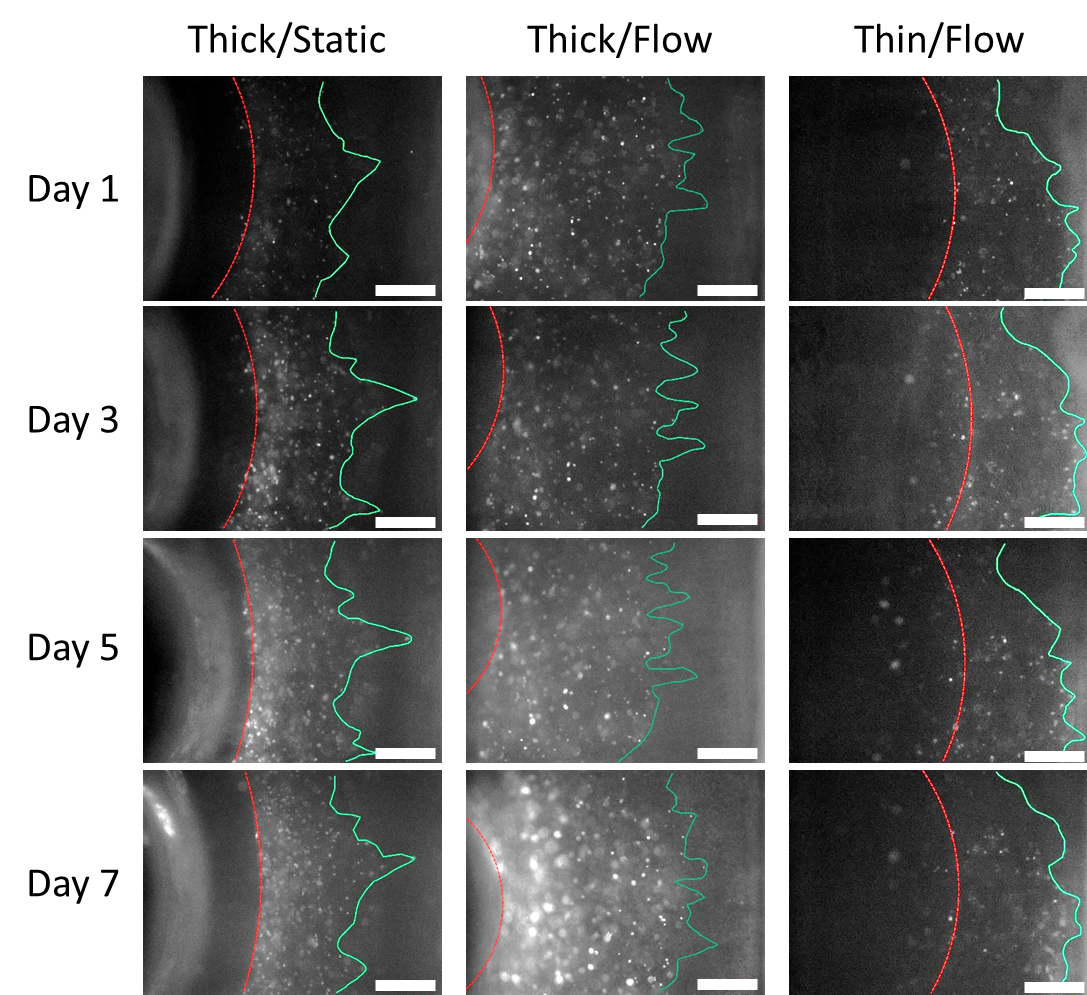


**Figure S3**. Fluorescence images of A549/GFP cells embedded in collagen and cultured for 7 days. Scale bar = 500 μm.


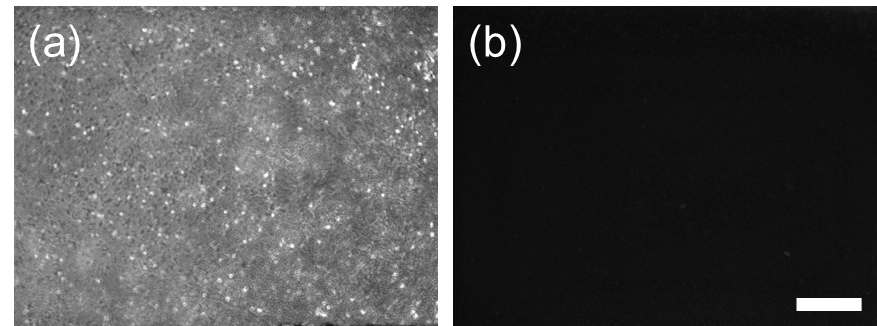


**Figure S4**. No A549/GFP cells were observed to migrate or penetrate the porous membrane. Microscopy observations using (a) bright field and (b) fluorescence. Scale bar = 100 µm.

**
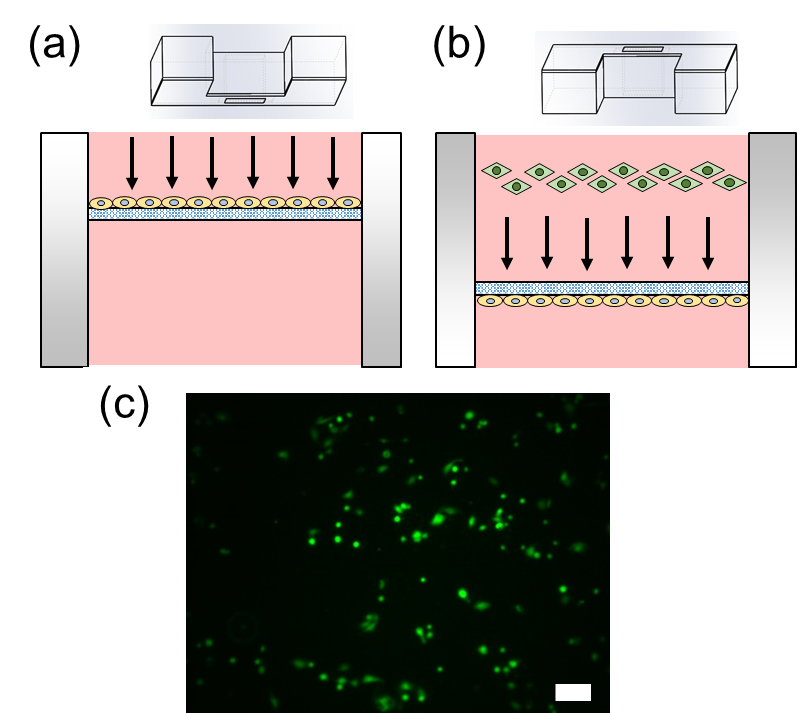
**

**Figure S5**. Co-culture procedure in the U-well. (a) HPMECs are seeded on the channel side of the membrane in advance. (b) After adhesion of the HPMECs, the A549-GFP cells can be seeded on the other side of the membrane (chamber side). (c) Live/dead staining assay demonstrates that the HPMECs cells are viable (green color) after one day of cell seeding. Scale bar = 100 µm.

**
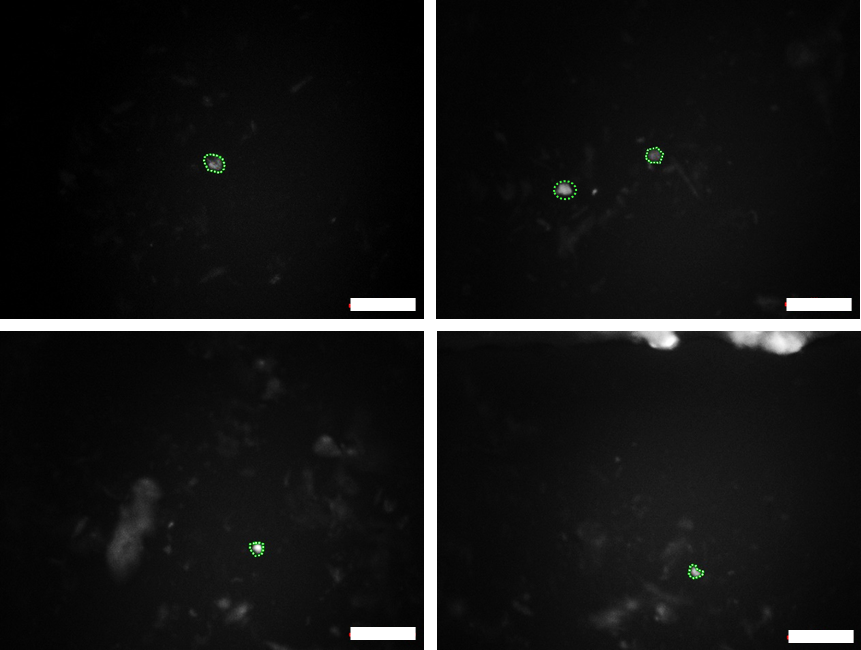
**

**Figure S6**. Fluorescence images of A549/GFP cells filtered on the membrane from the circulating medium after 17 days of culture. The live cells are circled using green dashed lines. Scale bar = 100 µm.


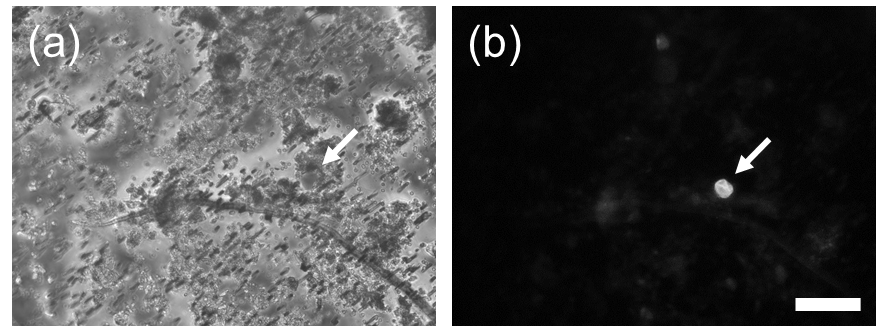


**Figure S7**. After 20 days of culture, the A549/GFP cells were collected from the circulating medium using the filter. Cell debris were observed under the microscope for (a) bright field and (b) fluorescence modes. The arrows indicate a live A549/GFP cell. Scale bar = 100 µm.


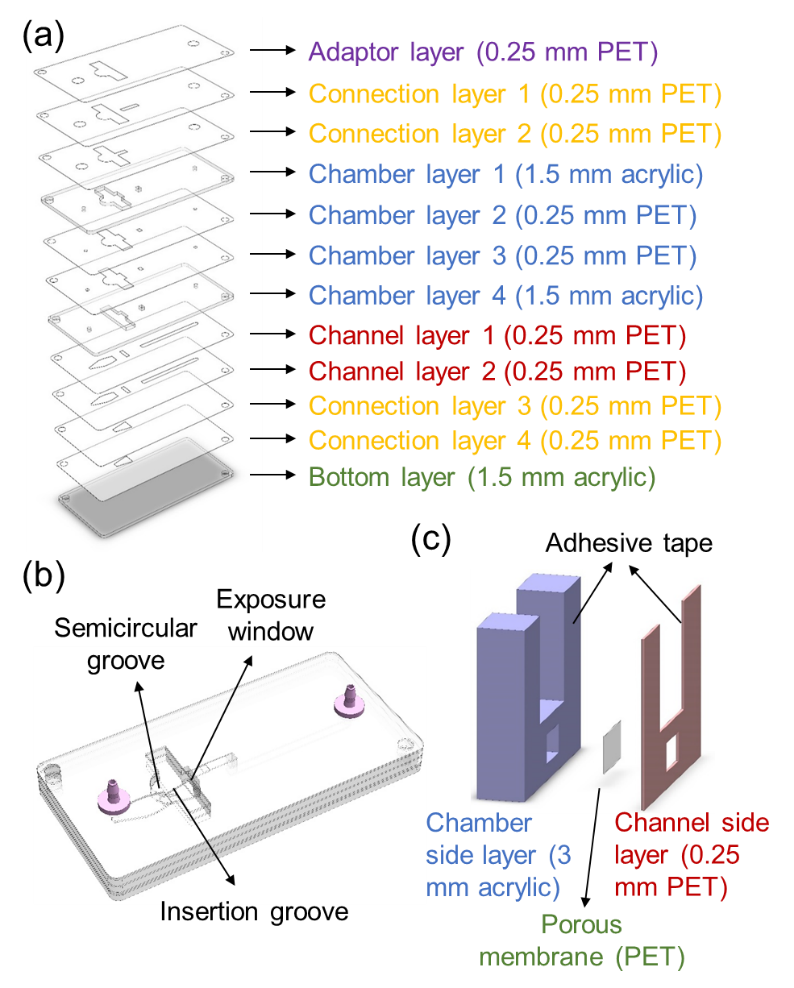


**Figure S8**. (a) Magnified view of the metastasis chip housing. The device is composed of 12 plastic layers. The layers can be distinguished into five groups as the adapter (purple), connection (yellow), chamber (blue), channel (red), and bottom (green) parts. (b) Metastasis chip housing assembled with tubing adaptors for pump connection. The semicircular groove is for storage of medium on the chamber side of the U-well, the insertion groove is for insertion of the U-well, and the exposure window is for exposing the cells to the medium from the circulating system. (c) The U-well is fabricated by sandwiching a porous membrane with a chamber side (thick) layer and a channel side (thin) layer using biocompatible adhesive tape.


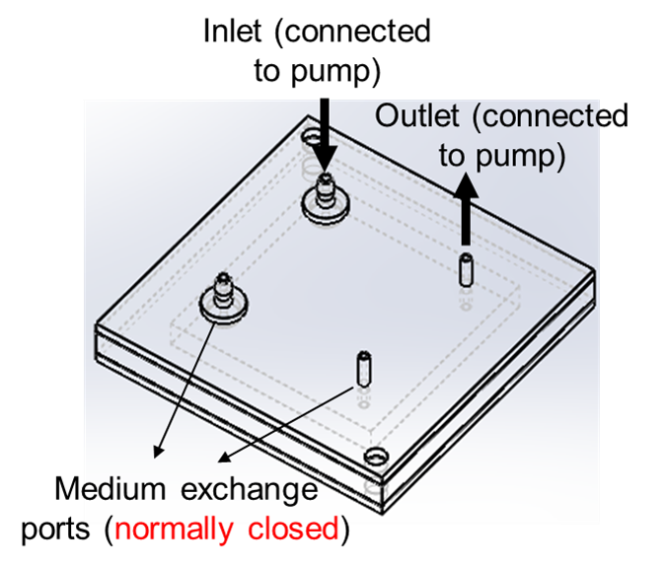


**Figure S9**. Schematic diagram of the medium reservoir. Outer dimensions = 50×50×7.5 mm and inner dimensions = 35×35×3.5 mm.


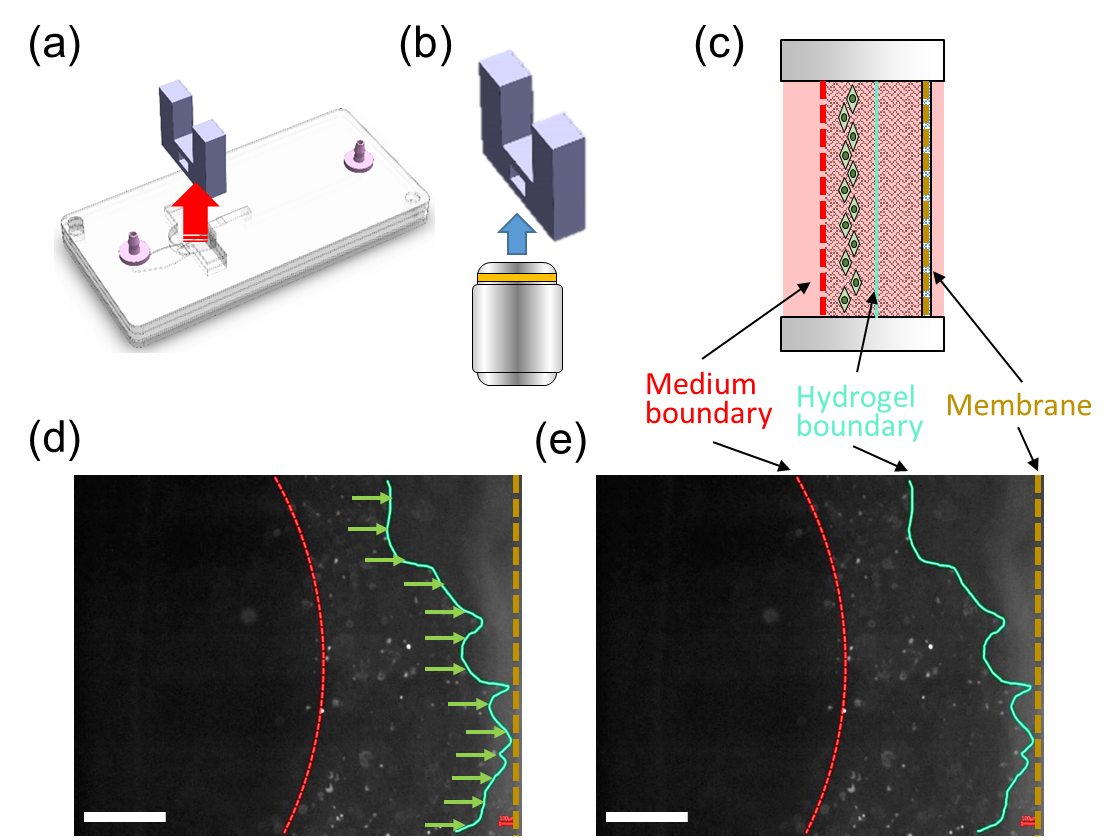


**Figure S10.** (a) The U-well can be pulled out from the metastasis chip housing. (b) The cells in the U-well can be monitored using an inverted microscope through the bottom side of the U-well. (c) Migration or proliferation area is defined between the medium boundary (red dashed line) and cell-free hydrogel boundary (green line). The boundary of the porous membrane is indicated by the brown dashed line. (d) Cell migration image. The green arrows show the direction of cell migration. (e) Cell proliferation image. Scale bar = 500 μm.

**
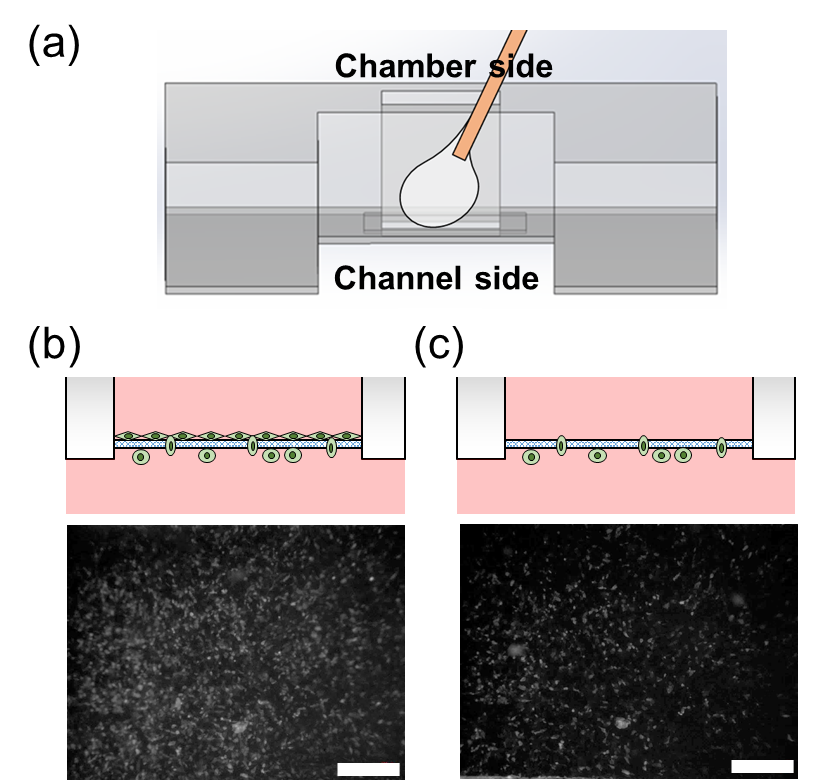
**

**Figure S11**. (a) Intravasated cells through the porous membrane can be quantified by removing the non-intravasated cells in the chamber side using cotton swabs. (b) Cells can be observed from the chamber and channel sides of the U-well before removal. (c) After removing the non-intravasted cells, the intravasated cells on the channel side of the U-well can be quantified by fluorescence microscopy. Scale bar = 500 μm.
